# The biological stress of early weaned piglets

**DOI:** 10.1186/2049-1891-4-19

**Published:** 2013-04-30

**Authors:** Joy M Campbell, Joe D Crenshaw, Javier Polo

**Affiliations:** 1 APC, Inc., 2425 SE Oak Tree Court, Ankeny, IA, USA

**Keywords:** Biological stress, Intestine, Pig, Weaning

## Abstract

Pigs experience biological stress such as physiological, environmental, and social challenges when weaned from the sow. The process of weaning is one of the most stressful events in the pig’s life that can contribute to intestinal and immune system dysfunctions that result in reduced pig health, growth, and feed intake, particularly during the first week after weaning. Technological improvements in housing, nutrition, health, and management have been used to minimize some of the adverse effects of weaning stress, but a greater understanding of the biological impact of stress is needed to improve strategies to overcome weaning stress. The focus of this review paper is to briefly describe how the biological stress associated with weaning impacts intestinal morphology, structure, physiology, and intestinal immune responses that can impact subsequent production efficiencies such as growth, intake, morbidity, and mortality.

## Introduction

Weaning pigs from the sow is one of the most stressful events in the pig’s life that can contribute to intestinal and immune system dysfunctions that result in reduced pig health, growth, and feed intake, particularly during the first week after weaning. Technological improvements in housing, nutrition, health, and management have been used to minimize some of the adverse effects of weaning stress, but a greater understanding of the biological impact of stress is needed to improve strategies to overcome weaning stress.

The focus of this review paper is to briefly describe how the biological stress associated with weaning impacts intestinal morphology, structure, physiology, and intestinal immune responses that can impact subsequent production efficiencies such as growth, intake, morbidity, and mortality.

### Stressors during weaning

The pig experiences significant physiological, environmental, and social challenges when it is weaned from the sow that can predispose the pig to subsequent diseases and other production losses. Weaning is one of the most stressful periods that results in intestinal, immunological, and behavioral changes. During this time, pigs are subjected to a number of stressors, such as, an abrupt separation from the sow, transportation and handling stress, a different food source, social hierarchy stress, co-mingling with pigs from other litters, a different physical environment (room, building, farm, water supply, etc.), increased exposure to pathogens, and dietary or environmental antigens. The piglet must adapt to all of these stressors rapidly in order to be productive and efficient. When the different stressors of weaning are too great for the pig to overcome, it can lead to poor performance and increased mortality.

### Feed intake and weaning

The gastro-intestinal system has multiple functions, such as, digestion and absorption of nutrients and electrolytes, maintenance of bodily fluid balance, secretion of digestive enzymes, mucin, immunoglobulins, and multiple other components, and to serve as a barrier for the host against harmful pathogens and antigens.

When the piglet is weaned, the piglet must adapt abruptly from highly digestible and palatable liquid milk from its mother that is equally spaced throughout the day to a solid dry diet that is less digestible and palatable. As a consequence, feed intake is usually reduced initially after weaning and the piglet becomes mal-nourished with reduced transient growth rate. As reviewed by Le Dividich and Sève [1], the extent and duration of reduced feed intake is variable. It is estimated that by the end of the first week post-weaning, metabolizable energy (ME) intake is about 60-70% of pre-weaning milk intake and that it takes approximately 2 wk post-weaning to achieve full recovery to the pre-weaning ME intake level. Spreeuwenberg et al. [2] evaluated the relationship between low feed intake with different diet compositions (lactose/protein ratios) and small intestinal barrier function. They reported during the first 4 d post-weaning, that diet composition was not as important a factor to maintain intestinal barrier function; but that continued low feed intake was more important to predispose the pig to intestinal barrier dysfunction. McCracken et al. [3] determined that low feed intake during the post-weaning period may contribute to intestinal inflammation and adversely affect villous height and crypt depth.

Growth performance is reduced with low feed intake. In general, pigs lose about 100–250 g body weight (BW) the first day after weaning, regardless of weaning age and recover this loss in BW by about 4 d post-weaning [1]. Tokach et al. [4] reported that weight gain in the first week after weaning impacts the total days to market (at approximately 110 kg BW). When pigs were gaining greater than 227 g/d during the first week after weaning, days to market was reduced about 6–10 d compared to pigs gaining 0 g/d to 150 g/d the first week.

Thus, it is important to get pigs eating and growing as soon as possible after weaning. It is difficult to prevent some of the decline in BW as the pigs move from sow’s milk to the starter diet. However, understanding the challenges and impact of low feed intake associated with weaning and the subsequent impact on performance can help the nutritionist better design diets by utilizing various feed ingredients and additives proven to increase feed intake and help the producer to use other management techniques that help to reduce the weaning stress.

### Structure and functional changes of intestine and weaning

Along with experiencing low feed intake, weaned pigs experience physiological changes in structure and function (enzyme activities and absorption or secretion) of the intestine. As reviewed by Pluske et al. [5], these physiological changes affect the absorptive capacity of the small intestine which can likely influence feed efficiency. Pluske et al. [5] and Boudry et al. [6] reported that weaning induces both acute and long-lasting structural and functional changes in the small intestine including shortening of the villi (villous atrophy) and an increase in crypt depth (crypt elongation) after weaning. Hampson et al. [7] demonstrated that villous height can rapidly decrease by about 25 to 35% of pre-weaning height within the first 24 h in pigs weaned at 21 d of age. The decrease in villous height continued until about 5 d after weaning, when the villi were approximately only half of the initial height. Unweaned groups showed only small changes in villous height. Crypt elongation was also evaluated with slower changes occurring over the first 11 d post-weaning. Additionally, Montagne et al. [8] reviewed several intestinal markers associated with weaning that may allow research to be conducted to reduce the physiological changes associated with weaning.

After weaning, pigs also experience reduced brush-border digestive enzyme activities [5]. Lalles et al. [9] reported reductions in lactase and amino-peptidase activity from d 2 to 15 post-weaning, while maltase was reduced for 2 d post-weaning, then increased d 8 to 15 post-weaning. Additionally pancreatic secretions had a transient decrease to d 15 after weaning, before trypsin and amylase activity began to increase. Alkaline phosphatase, an enzyme that plays a role in detoxification of pathogenic bacterial lipopolysaccharide endotoxin and impacts intestinal inflammation [10], is also reduced in early weaned pigs [11]. These alterations can impact the ability of the small intestine’s digestive, absorptive, and secretory capacity and ultimately the intestinal barrier function, which may contribute to post-weaning diarrhea.

### Inflammation associated with weaning

Beyond the compromised digestive and absorptive capacity, consequences associated with weaning also induce a deleterious effect on intestinal barrier function [2,6,12]. The epithelial layer of the intestinal lumen serves as the body’s first line of defense for protecting the pig from various harmful microorganisms, toxins, or antigens that reside within the lumen of the small intestine. When the intestinal barrier is disrupted, the result is increased permeability that allows toxins, bacteria, or feed-associated antigens to cross the epithelium resulting in inflammation, mal-absorption, diarrhea, and reduced growth and production.

Nabuurs et al. [13] evaluated the effects of weaning and *Escherichia coli* infection on absorption capacity of the small intestine in pigs. Pigs were either weaned at 30 to 32 d of age or unweaned by remaining on the sow, while *Escherichia coli* infection consisted of infecting segments of the small intestine using perfusion procedures. In unweaned non-infected pigs, no differences were noted in net absorption of fluid, potassium or chloride. However, pigs that had been weaned but not infected with *Escherichia coli* had less fluid absorption on d 4, 7, and 14, while sodium and chloride absorption was less on d 4 and 7 in the intestinal segments compared to segments from unweaned non-infected pigs. In the *Escherichia coli* infected intestinal segments of weaned pigs, fluid absorption was less on d 11 and 14, while sodium and potassium on d 11, and chloride absorption on d 4 and 11 was less than that of infected intestinal segments from unweaned pigs. Furthermore, *Escherichia coli* infected weaned pigs had greater net reduced absorption compared to infected unweaned pigs. The authors concluded that after weaning the net absorption of fluid and electrolytes is temporarily decreased which may contribute to diarrhea.

Studies conducted at North Carolina State University have investigated the effects of stress-induced intestinal damage associated with a weaning stress model. Moeser et al. [12] evaluated intestinal dysfunction in 19 d old weaned pigs compared to unweaned pigs. Twenty–four h after weaning, the pig’s intestinal barrier function was evaluated for secretory activity by transepithelial resistance (TER) and intestinal permeability by paracellular mannitol flux in the jejunum and colon. In both the jejunum and colon, weaned pigs had greater secretory activity and intestinal permeability than unweaned pigs. This research corresponds with Boudry et al. [6], who demonstrated a transient reduction in jejunal TER, but not in the colon. Moeser et al. [12] also evaluated stress hormones and intestinal barrier function over 7 d post-weaning. They reported increased serum corticotrophin-releasing factor (CRF) and cortisol in weaned pigs indicating that weaning induces activation of the stress pathways which may be mediating the intestinal dysfunction. Subsequent studies by Moeser et al. [14] demonstrated that weaning age can affect the intestinal stress response in the pig. Finally, Smith et al. [15] utilizing the weaning stress model evaluated the effects of early weaning stress on intestinal barrier function and intestinal health. Pigs from 5 different weaning ages (15, 18, 21, 23, and 28 d of age) were utilized. At 35 d of age, all pigs were evaluated for secretory activity and intestinal permeability in the jejunum. The results indicate that as weaning age was incrementally increased, improvements in intestinal barrier function were observed as indicated by improved TER and lower permeability as measured by mannitol and inulin flux. To evaluate if the intestinal dysfunction was sustained, 15 or 28 d old weaned pigs were evaluated after 9 wk of age. The results were similar to previous observations; earlier weaned pigs had a reduced TER and increased permeability. Thus, the research demonstrated that the stress resulting from weaning induces a breakdown in intestinal barrier function associated with increased permeability and mucosal inflammation and that weaning age can impact present and future mucosal barrier function [12,14,15].

Other immunological responses that occur during the weaning process are alterations in pro-inflammatory cytokines. Pro-inflammatory cytokines have an influence on intestinal integrity and epithelial function as it relates to permeability and transport of nutrients [16]. Pié et al. [17] evaluated gene expression of pro-inflammatory cytokines such as IL-1β, IL-6, and TNF-α during the weaning process. They utilized 28 d old pigs and measured gene expression over 8 d post-weaning. The research demonstrated that weaning is associated with an up-regulation of pro-inflammatory cytokines. Pié et al. [17] reported increased TNF-α expression initially by d 1 in the proximal and mid intestine followed by increases in the distal small intestine and proximal colon from d 2 to 8. Thus, indicating that weaning is associated with an early up-regulation of genes of pro-inflammatory cytokines that may contribute to functional disorders resulting in reduced subsequent performance and play a role in post-weaning diarrhea.

Regulation of metabolism may also be altered in response to weaning when inflammation associated with increased expression of pro-inflammatory cytokines occurs. Pro-inflammatory cytokines regulate both immune function and growth or metabolic processes [18,19]. Thus, weaning stress impacts both structural alterations and active immune responses. The intestine is a major site for amino acid oxidation, net synthesis, and utilization of amino acids for protein synthesis as reviewed by Burrin et al. [20]. Alterations in amino acid metabolism occur after weaning [8], which may impact protein synthesis and subsequent tissue deposition. Williams et al. [21-23] demonstrated that when the immune system is activated growth, feed intake, feed efficiency, and lean tissue deposition is reduced. Therefore, the reduction or mitigation of post-weaning stress and subsequent effects in the structural changes of the intestine and activation of the inflammatory immune response is critical for improving swine performance from weaning to market.

To counter or reduce the negative effects associated with weaning and pro-inflammatory cytokine activation, heat shock proteins may play a role in mitigating the effect. Heat shock proteins have various cytoprotective functions related to various kinds of stress responses as reviewed by Feder et al. [24]. Heat shock proteins are produced by epithelial cells in response to heat stress to help protect cells under stress situations. David et al. [25] utilized 21 or 28 d old pigs to evaluate the impact of weaning on intestinal heat shock protein patterns. The heat shock proteins 27 and 70 were transiently increased in the stomach and duodenum between 6 to 12 h after weaning and in the mid-jejunum, ileum, and colon between 24 to 48 h after weaning, while in the ileum the heat shock proteins were transiently decreased 36–38 h. In unweaned pigs, heat shock protein expression was similar in all segments of the intestine. The factors that impact the spatial modulation are unknown. However, the increase in heat shock proteins associated with weaning may help to reduce the negative effects of inflammation and weaning stress by their cytoprotection mechanism that involves both inhibition of pro-inflammatory cytokines and cellular proliferation [26]. Further research is needed to understand modulation of heat shock proteins and their beneficial effects in the intestinal tract.

Research into other methods such as increasing feed intake, reducing disease exposure, and utilization of nutritional ingredients such as glutamine, spray-dried plasma, colostrum, arginine, pre- and probiotics, n-3 long chain polyunsaturated fatty acids, and n-6 linoleic acid that may reduce negative effects associated with weaning is needed to further improve production efficiencies. These and other technologies are beyond the scope of this paper.

## Conclusions

Biological alterations in metabolism, immune system, and intestinal functions occur during and immediately after weaning that may have both short and long-term effects on subsequent pig growth and health, regardless of age of pig at weaning. It is critical that swine producers utilize appropriate health, nutrition, and management strategies to minimize the adverse effects of weaning stress and to improve swine productive measures all the way to market weight.

## Competing interest

The authors declare they have no competing interest.

## Authors’ contributions

JMC composed the manuscript and JDC and JP reviewed and provided input. All authors read and approved the final manuscript.
